# Chronic pharyngitis and cervical spondylosis risk: A bidirectional Mendelian randomization study

**DOI:** 10.1097/MD.0000000000041678

**Published:** 2025-02-21

**Authors:** Yuwei Li, Xiaoxi Li, Xinya Yu, Yunchun Xu, Yunpeng Su, Le Guo

**Affiliations:** a Department of Medical Microbiology and Immunology, School of Basic Medical Sciences, Dali University, Dali, Yunnan, P.R. China; b School of Clinical Medicine, Dali University, Dali, Yunnan, P.R. China.

**Keywords:** causal inference, cervical spondylosis, chronic pharyngitis, Mendelian randomization

## Abstract

Cervical spondylosis (CS) is the most prevalent degenerative disease among the elderly. Chronic pharyngitis (CP) has been reported as a contributing factor to CP. However, the causal relationship between CP and CS has not yet been established. This study aims to investigate the potential link between CP and CS. A bidirectional Mendelian randomization (MR) analysis was performed using genome-wide association studies. Single nucleotide polymorphisms for each trait were identified as instrumental variables. Several sensitivity analyses, including inverse-variance weighted (IVW) method, weighted median method, MR-Egger regression, MR-PRESSO, and outlier test, were conducted to validate MR assumptions. The analysis showed that CP influences the risk of CS, as evidenced by the IVW method (odds ratio [OR]: 1.183, 95% CI: 1.091–1.282, *P* < .001), MR-Egger (OR: 1.65, 95% CI: 0.966–1.405, *P* = .124), and weighted median method (OR: 1.156, 95% CI: 1.031–1.297, *P* = .015). Conversely, the impact of CS on CP incidence was not strongly supported, as shown in the IVW (OR: 1.083, 95% CI: 1.019–1.152, *P* = .009), MR-Egger (OR: 0.910, 95% CI: 0.752–1.101, *P* = .337), and weighted median analyses (OR: 1.060, 95% CI: 0.972–1.157, *P* = .182). The findings suggest that CP may increase the risk of developing CS.

## 1. Introduction

Cervical spondylosis (CS) is characterized as a degenerative disorder that is noninflammatory in nature. The primary lesion typically involves disc degeneration near the amphiarthrodial joint, formed by adjacent vertebral bodies and the intervening disk.^[1,2]^ Age plays a crucial role in cervical disc degeneration, with the majority of affected individuals being over 50 years old. Common findings include mucosal congestion, edema, and hypertrophy in the pharynx, often accompanied by a history of recurrent acute pharyngitis. The manifestations of CS vary depending on the stage of the pathological process and the location of neural compression. Diagnosis usually requires confirmation through a cervical X-ray and, if necessary, myelography.^[3]^ Radiological investigations such as X-ray, CT, or MRI of the cervical spine often reveal alterations in physiological curvature, instability or osteophyte formation, hyperplasia of the uncinate joint, ligament calcification, and sagittal stenosis of the spinal canal.^[1]^ However, surgical intervention for CS patients demands careful consideration of factors such as the patient’s age, medical history duration, whether the disc herniation is single or multiple, and cardiovascular system status. Hence, early diagnosis of CS high-risk groups has gained increasing significance in CS research.

Traditional Chinese medicine posits that recurrent pharyngitis might be a contributing factor to CS, termed “pharyngitis-related CS.” Chronic pharyngitis (CP) is characterized by diffusive inflammation of the pharyngeal mucosa, submucosa, and pharyngeal lymphoid tissues. It is a common ailment, accounting for about 10% to 20% of throat diseases.^[4]^ In the United States, roughly 10 million outpatients receive treatment for CP annually.^[5,6]^ In China, the prevalence of CP is notably high, attributed to dietary habits, environmental factors, and antibiotic misuse, with a reported incidence of 78.65% in physical examination populations.^[7]^ CP is categorized into chronic nasopharyngitis and tonsillopharyngitis. Its primary clinical symptoms include recurrent hoarseness, irritating cough, sore throat, and difficulty in swallowing. To date, limited studies and case reports have investigated the contribution of CP to CS, and there is a lack of randomized controlled trials or cohort studies to confirm their correlation. Consequently, the causal role of CP in the development of CS remains unclear. This study hypothesizes that CP may have a positive causal influence on the development of CS.

Mendelian randomization (MR) is a genetic approach using single nucleotide polymorphism (SNP) as an instrumental variable to investigate potential causal relationships between risk factors and outcomes.^[8,9]^ This method strengthens inferences about the causal nature of associations by reducing the potential for confounding in conventional observational studies and eliminating reverse causality. In this study, the bidirectional MR approach was employed to explore the causal relationship between CP and CS, offering a novel clinical perspective for the diagnosis and treatment of CS.

## 2. Materials and methods

### 2.1 Study design

In this investigation, a bidirectional MR approach was employed to explore the causal link between CP and CS using genetic instrumental variables (IVs). Utilizing data from the FinnGen database GWAS, 21 statistically significant SNPs associated with CP were identified for positive MR, considering CS as the outcome. Conversely, reverse MR utilized 51 SNPs linked to CS as IVs, with CP as the outcome (Supplementary Files 1 and 2, Supplemental Digital Content, http://links.lww.com/MD/O430, which presents the results of the sensitivity analysis).

## 3. Chronic pharyngitis and cervical spondylosis genome-wide association studies

Table 1 presents a summary of the data sources. The pooled data for GWAS related to CP and CS were extracted from the FinnGen database R9 (https://www.finngen.fi/en/, last accessed on November 10, 2023).^[10]^ This included 10,868 cases involving chronic rhinitis, nasopharyngitis, and pharyngitis, alongside 283,342 control cases (https://r9.risteys.finngen.fi/endpoints/J10_CHRONRHINITIS). The mean age at first publication was 49.27 years. The data set for CS comprised 13,394 cases with cervical disc disorders and 270,964 control cases, with an average age of first onset at 49.23 years, and 1165 deaths due to CS during the 1998 to 2019 follow-up period (https://r9.risteys.finngen.fi/endpoints/M13_CERVICDISC). To minimize age-related bias, 2 GWAS with similar average ages were selected as exposure and outcomes for CS.

**Table 1:** Details of the GWASs included in the MR.

Variable	Sample size	SNP number	Gender	Average age	Race	Yr
Chronic pharyngitis	294,210	20,168,003	Both sexes	49.27	European	2021
Cervical spondylosis	284,358	20,167,547	Both sexes	49.23	European	2021

GWASs = genome-wide association studies, MR = Mendelian randomization, SNPs = single nucleotide polymorphisms.

## 4. Selection of instrumental variables

For SNPs to serve as IVs in examining the causal relationship between exposure and outcomes, 3 criteria must be met in this study: IVs must exhibit a strong correlation with exposure. IVs should not be associated with confounders in the exposure-outcome relationship. IVs must influence the outcome solely through exposure, without other pathways. Thus, SNPs significantly associated with exposure were selected at the genome-wide significance level (*P* < 5 × 10^−8^, linkage disequilibrium *r*^2^ < 0.001, genetic distance = 10,000 KB). The *F*-statistics, calculated by the formula *F* = (N-*K*-1/*K*) × (*R*^2^/[1-*R*^2^]), were employed to eliminate the bias from weak IVs, with a threshold of *F* > 10 indicating strong IVs. In this formula, N denotes the sample size of the dataset, β represents the effect size of SNP on exposure, SE is the standard error of β, and EAF denotes the effect allele frequency, with *R*^2^ = 2 × (1-EAF) × EAF×(β)^2^.^[11]^

To encompass more SNPs contributing to CP status, a relaxed threshold (*P* < 5 × 10^−6^, *r*^2^ < 0.1) was applied, identifying 23 SNPs. The *F*-statistics, exceeding the conventional threshold of 10, suggested these instruments had robust predictive potential for CP. For the reverse MR analysis, 51 SNPs strongly correlated with CS were identified (*P* < 5 × 10^−6^, *r*^2^ < 0.001, genetic distance = 10,000 KB, *F* > 10).^[12]^ Additionally, the PhenoScanner database (www.phenoscanner.medschl.cam.ac.uk) was consulted to determine if these SNPs were linked to potential risk factors such as body mass index, obesity, long-term bow strain. SNPs associated with any of these potential confounders at the genome-wide significance level were excluded.

## 5. Mendelian randomization analyses

In the MR analyses following harmonization of the effect alleles in the GWASs of CP, SNPs rs12155833 and rs7801065 were excluded. Various MR methods were employed to determine the MR estimates of CP for CS, including the random-effect inverse-variance weighted (IVW), weighted median, and MR-Egger. These approaches were chosen as they operate under different assumptions regarding horizontal pleiotropy.^[13]^ IVW was the primary method, while MR-Egger and weighted median served to refine the IVW estimates. Although these methods may provide more robust estimates in various scenarios, they tend to be less efficient, as indicated by wider CIs. The MR-Egger regression employs the reciprocal of the outcome variance as the weight to assess and calculate the effect of exposure on the outcome.^[14,15]^ The weighted median method relies on the assumption that at least half of the IVs are valid for analyzing the causal association between exposure and outcome. The odds ratio (OR) was utilized to evaluate the potential causal relationship between CP and CS. Cochran *Q*-test assessed heterogeneity among individual genetic variants. Additionally, MR-Egger intercept test and MR-PRESSO were used for detecting and correcting horizontal pleiotropy.^[16–18]^ A leave-one-out analysis was conducted to determine if the MR estimate was influenced or biased by any single SNP. The reverse MR analysis applied the same methodology as above. Statistical power was calculated using an online web tool (https://sb452.shinyapps.io/power/).^[19]^ All analyses were conducted using the TwoSampleMR (version 0.5.6) and MR-PRESSO (version 1.0) packages in R (version 4.6.1).

## 6. Result

### 6.1 Causal effect from CP to CS

**Figure 1.**
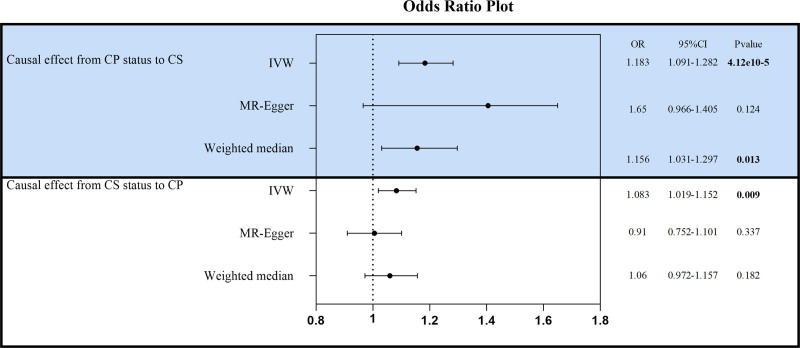
Bidirectional MR study of association between CP and CS. CI = confidence interval, CP = chronic pharyngitis, CS = cervical spondylosis, IVW = inverse-variance weighted, MR= Mendelian randomization, OR = odds ratio.

**Figure 2.**
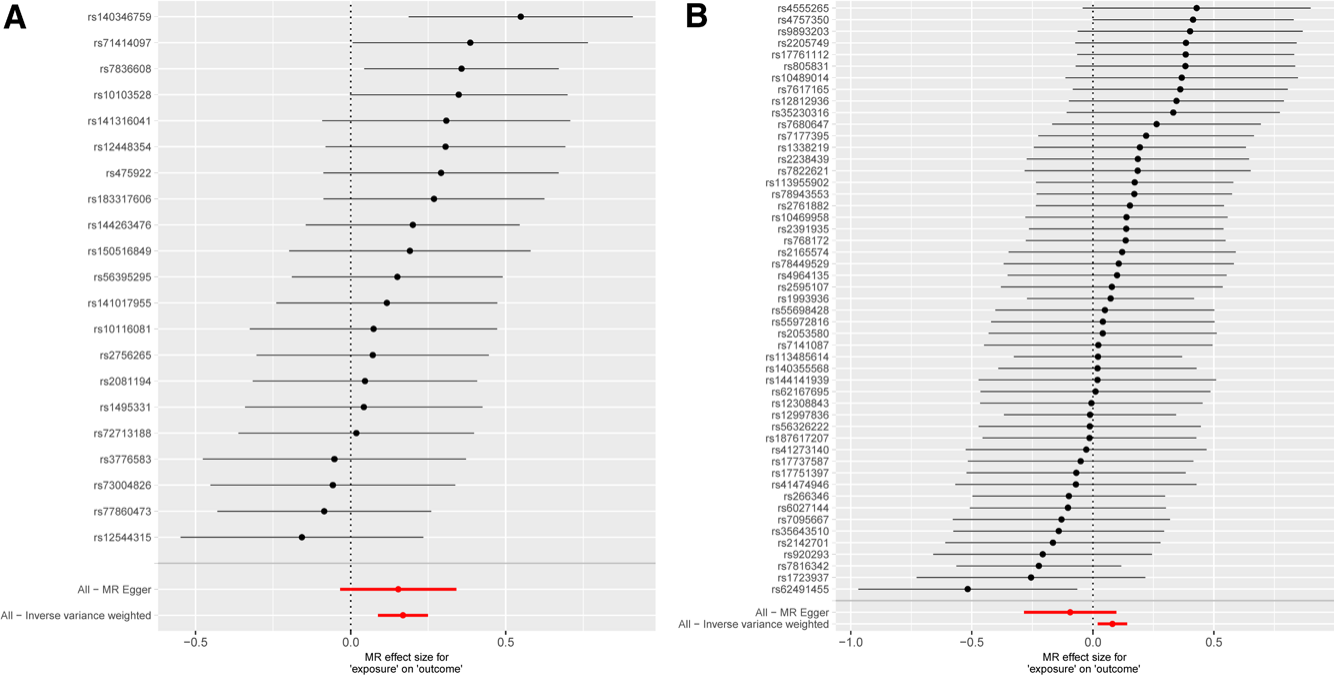
(A) Forest plot for forward MR of the association between CP and CS. (B) Forest plot for reverse MR of the association between CS and CP. CP = chronic pharyngitis, CS = cervical spondylosis, MR = Mendelian randomization.

**Figure 3.**
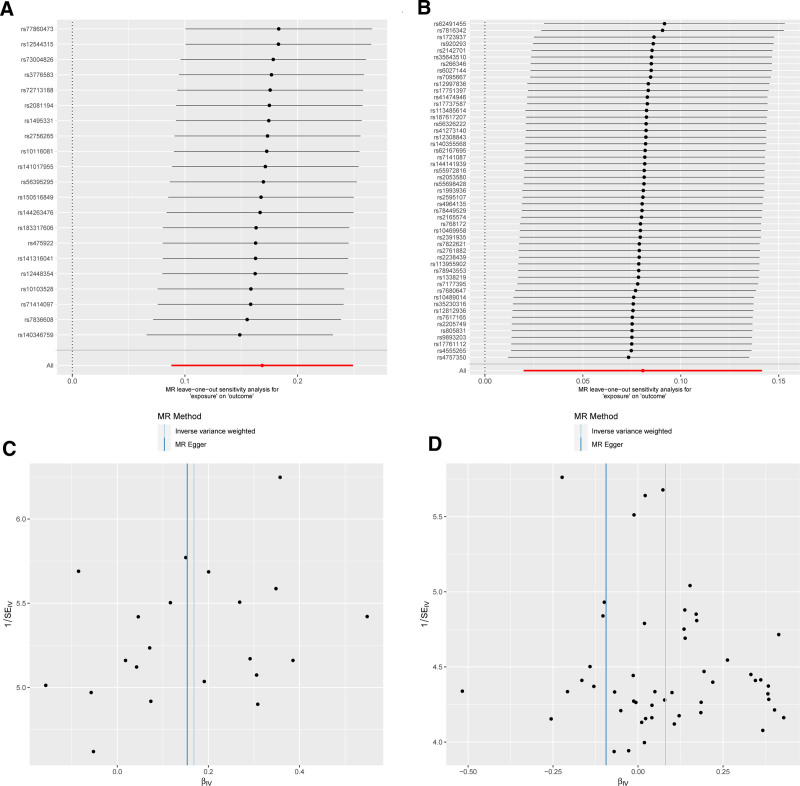
(A) Forest plot for forward MR of the association between CP and CS after removal of SNPs 1 by 1. (B) Forest plot for reverse MR of the association between CS and CP after removal of SNPs 1 by 1. (C) Scatter plot for forward MR of the association between CP and CS. (D) Scatter plot for reverse MR of the association between CS and CP. CP = chronic pharyngitis, CS = cervical spondylosis, MR Mendelian randomization; SNPs single nucleotide polymorphisms.

In this investigation, 21 SNPs linked to CP were employed as IVs for MR analysis. The findings revealed that patients with CP had 18.3% heightened risk of developing CS (OR_IVW_: 1.183, 95% CI: 1.091–1.282, *P* < .001). Additionally, MR-Egger regression (OR: 1.65, 95% CI: 0.966–1.405, *P* = .124) and the weighted median method (OR: 1.156, 95% CI: 1.031–1.297, *P* = .013) were applied (Figs. 1 and 2). The Cochran *Q*-test indicated no heterogeneity (*P* = .542), and no horizontal pleiotropy of IVs was identified through Egger intercept (*P* = .862) and MR-PRESSO methods (*P* = .588), suggesting the IVs did not significantly affect the outcome through any means other than exposure (Supplementary File 3, Supplemental Digital Content, http://links.lww.com/MD/O430, which presents the results of the sensitivity analysis). The robustness of the MR analysis results was confirmed by a leave-one-out sensitivity analysis (Fig. 3A). Funnel plots confirm a symmetric distribution of causal effects obtained from each IV (Fig. 3C).

### 6.2 Causal effect from CS status to CP

The reverse MR analysis utilized 51 SNPs associated with CS as IVs. The outcomes indicated a potential influence of CS on CP (OR_IVW_: 1.083, 95% CI: 1.019–1.152, *P* = .009), although MR-Egger showed contrasting results (OR: 0.910, 95% CI: 0.752–1.101, *P* = .337) (Figs. 1 and 2). The Cochran *Q*-test yielded a *P*-value of .845. The *P*-values from the Egger intercept and MR-PRESSO methods were both > .05 (Supplementary File 3, Supplemental Digital Content, http://links.lww.com/MD/O430, which presents the results of the sensitivity analysis), and the leave-one-out sensitivity analysis results were stable (Fig. 3B). Funnel plot shows a symmetrical distribution of points on the left and right sides of the IVW line, indicating that the results are unlikely to be affected by potential bias (Fig. 3D).

## 7. Discussion

In this study, we utilized bidirectional MR to investigate whether CP exerts a causal effect on the incidence of CS. Historically, most research has identified age, gender, and occupation as contributing factors for CS.^[20]^ However, only a limited number of epidemiological studies have indicated CP as a risk factor for CS, without clearly establishing a causal link due to ambiguous chronological sequencing. An observational study examined the association between throat lumps and cervical degenerative disease by correlating clinical symptoms with imaging features.^[21]^ Additionally, previous observational studies struggled to eliminate biases from confounding risk factors. By implementing MR methods in our research, we were able to determine causality beyond such biases.

In this study, IVW method, MR-Egger regression and weighted median method were used for 2-sample MR analysis. IVW assumes that all IVs are valid, i.e. they affect the results only through exposure and have no other direct effects. Therefore, we must ensure that these SNPs are not pleiotropic when using IVW, otherwise the results will be greatly biased. In MR-Egger hypothesis, we consider the existence of the intercept term and use it to evaluate pleiotropy.^[13]^ Weighted median: allows some IVs to have horizontal pleiotropy. Based on the complementary role of the 3 methods in addressing horizontal pleiotropy, this study uses these 3 methods to comprehensively evaluate the robustness of MR Results under different assumptions. The positive MR analysis indicated that CP is associated with an increased risk of CS, and this was further corroborated by sensitivity analysis, affirming a positive causal relationship between the 2 conditions. This suggests that the incidence of CS should be closely monitored in patients with CP. An observational study evaluating the association between throat mass and cervical degenerative disease by clinical symptoms and associated radiographic features found that patients with throat mass symptoms had significantly more total bone spurs, C45 (OR: 2.582, 95% CI: 1.428–4.669, *P* = .002), C56 (OR: 9.898, 95% CI: 3.176–30.845, *P* < .001), and C67 (OR: 3.102, 95% CI: 1.616–5.957, *P* < .001) were the major loci.^[21]^ It is suggested that CP is highly correlated with cervical osteophytes, which is consistent with the results of our analysis.

It is often observed that patients with CS are accompanied by recurrent pharyngitis,^[22]^ and this study confirmed for the first time that CP is 1 of the risk factors for CS. However, the specific mechanism has not been elucidated. With recent findings revealing multiple inflammatory mediators in degenerative intervertebral discs, inflammation is now recognized as a key pathological mechanism in CS.^[23]^ Numerous studies have linked disc degeneration with cytokine imbalance, highlighting the role of IL-1b, IL-6, and TNF-a in exacerbating this degeneration.^[24,25]^ Marshall research further supports this, showing elevated levels of histamine, IL-1b, TNF, and PGE2 in herniated intervertebral discs compared to normal discs, correlating with the degree of degeneration.^[26]^ Therefore, previous studies generally believe that the inflammation caused by pharyngitis affects the course of CS. Considering the anatomical proximity of the throat to the cervical disc and their shared lymphatic connections, inflammation in the throat can have implications for the cervical region. Inflammatory processes in the throat can allow microorganisms such as bacteria and viruses to travel to the retropharyngeal lymph nodes via the deep superior and inferior cervical lymph nodes. These pathogens can then spread to the area around the atlanto-occipital joint, collected by the retropharyngeal lymph nodes, impacting neck muscles, ligaments, and joint capsules, ultimately leading to decreased muscle tension in the neck. Pan et al found that with the prolongation of pathological cycle, the pathological morphology of pharyngitis mucosa and cervical intervertebral disc in rabbit model showed chronic inflammatory changes and gradually worsened. IL-1, IL-6, and PGE2 increased with the duration of CP.^[27]^ Furthermore, in our SNP screening, rs12448354 was found to be highly correlated with lymphocyte count. Based on these findings, we propose that chronic throat infections and the resultant inflammation could be a contributing mechanism to the development of CS.

In our reverse MR Analysis, MR-Egger regression and weighted median method were not statistically significant. As we said before, the IVW method assumes that the intercept is 0, and does not consider the gene pleiotropy of the included IVs. However, MR-Egger regression is not limited to the intercept being 0, but is modified on the basis of IVW, and the influence of gene pleiotropy on the result is also considered (Supplementary File 4, Supplemental Digital Content, http://links.lww.com/MD/O430, which shows the scatter plot). Therefore, inconsistent estimates are given that there is no causal association between reverse MR. In addition, the OR value obtained by the MR-egger regression is < 1, which deviates from the direction indicated by the IVW and weighted median methods. We caution that there is insufficient evidence to support a significant causal association between exposure factors (CS) and outcome (CP) in reverse MR. In summary, although the IVW method showed positive results, due to the inconsistent results of other methods, we cannot consider the reverse MR Results to have a clear positive significance. Further research and more tests may be needed in the future to clarify the true relationship between CS and CP.

However, our study also had several limitations. Firstly, it was confined to individuals of European descent, thus the findings may not be generalizable across different ethnicities, countries, and regions. Secondly, the lack of detailed clinical data precluded a thorough causal analysis within subgroups. Population stratification is a well-known source of confounding bias in high-throughput genomic data. In MR, we can only rely on alternative analyses of modified identification assumptions (e.g., MR-Egger, MR-PRESSO, Cochran *Q*-test, and leave-one-out analysis). These methods have been shown to be useful in partially alleviating these problems, but residual biases may still exist.^[28]^ In terms of 2-sample MR, ideally, there should be no overlap between the datasets used for exposure and outcome. However, both the exposure and outcome GWAS data in our study were sourced from the FinnGen database, making complete non-overlap challenging. In our study, the proportion of overlapping participants was 3.08% (Supplementary File 5, Supplemental Digital Content, http://links.lww.com/MD/O430, which illustrates the rate of population overlap between the 2 samples). According to Burgess simulation, this overlap ratio is considered within an acceptable range.^[29]^

## 8. Conclusion

In summary, our study suggests that patients with CP are at a causally increased risk of developing CS compared to the general population. Consequently, clinicians should be vigilant in monitoring for the development of CP in patients who have a history of long-term recurrent pharyngitis.

## Acknowledgments

We want to acknowledge the participants and investigators of FinnGen study.

## Author contributions

**Conceptualization:** Yuwei Li, Le Guo.

**Formal analysis:** Yuwei Li.

**Methodology:** Yuwei Li, Xiaoxi Li.

**Visualization:** Yuwei Li, Xinya Yu, Yunchun Xu, Yunpeng Su.

**Writing – original draft:** Yuwei Li.

**Writing – review & editing:** Yuwei Li, Xinya Yu, Le Guo.

## Supplementary Material


