# Supplementary material for: Chronic pharyngitis and cervical spondylosis risk: A bidirectional Mendelian randomization study
Source: Medicine (Baltimore). 2025 Feb 21;104(8):e41678. doi: 10.1097/MD.0000000000041678 (PMC11856965; doi:10.1097/MD.0000000000041678)
Supplement: Supplementary file 1 [file medi-104-e41678-s001.docx]

**File1**. Forward Mendelian randomization: Genetic variants used in the analyses investigating a causal impact of genetically predicted chronic pharyngitis and cervical spondylosis.

**File 2.** Reverse Mendelian randomization: Genetic variants used in the analyses investigating a causal impact of genetically predicted cervical spondylosis and chronic pharyngitis.

**File 3.** Results of the MR-PRESSO global and MR-Egger intercept tests for detecting horizontal and directional pleiotropy, respectively, and between SNP-heterogeneity based on the two-sample radial Mendelian randomization framework.

|  | **Heterogeneity trait** | | **Pleiotropy trait** | | | |
| --- | --- | --- | --- | --- | --- | --- |
|  | Cochran´s Q | *p*-value | egger-intercept | *p*-value | MR-PRESSO | *p*-value |
| **Forward MR** | 18.683 | 0.542 | 1.65E-03 | 0.862 | 20.623 | 0.588 |
| **Reverse MR** | 39.921 | 0.845 | 0.014 | 0.063 | 41.539 | 0.846 |

*
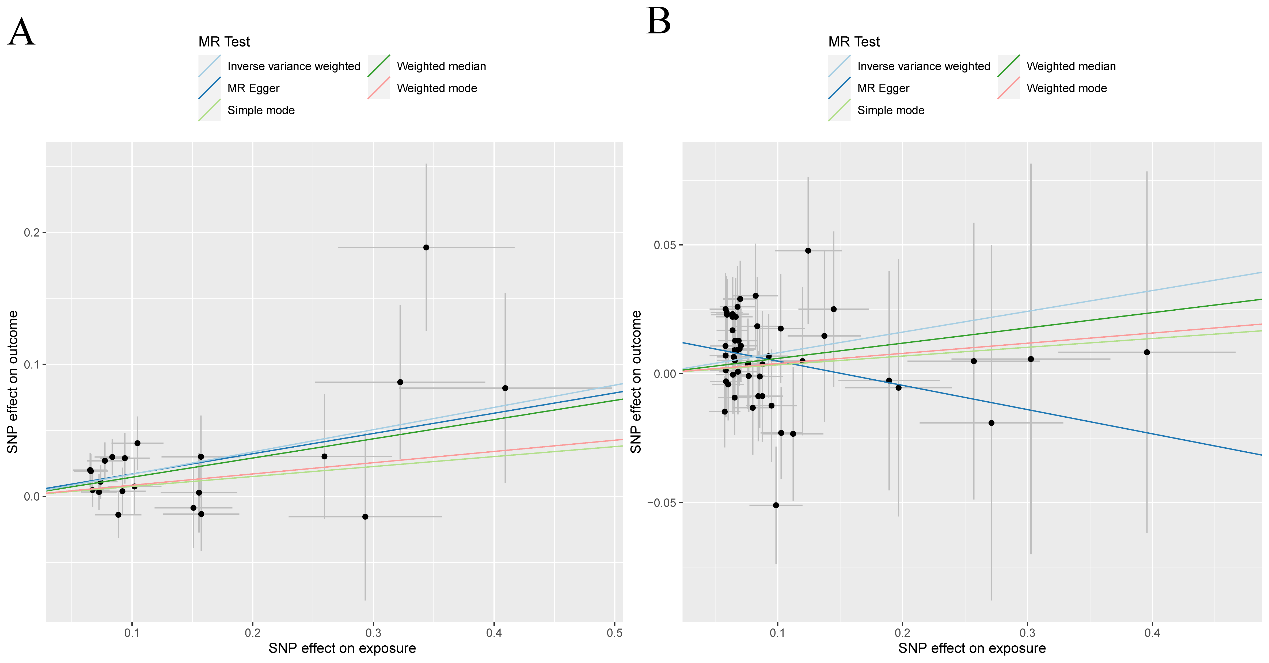
*

**File 4.** （A）Forward Mendelian randomized scatter plot.(B) Reverse Mendelian randomized scatter plot.

ONRHINITIS)


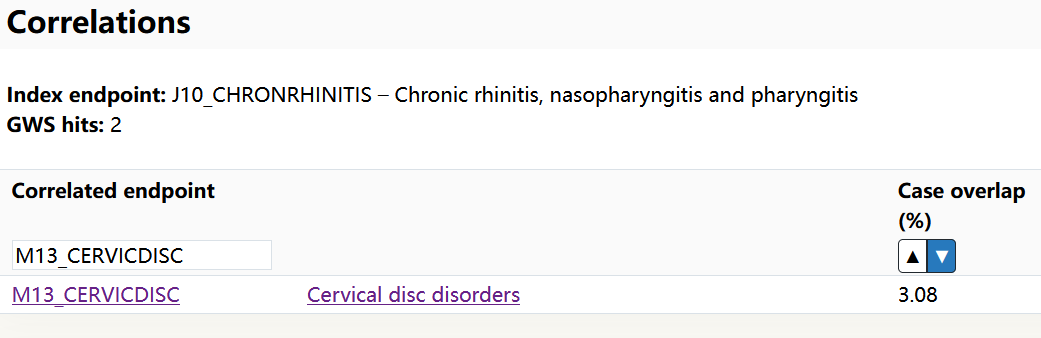


**File 5.** Case overlap rate of exposure and outcome. This data is provided by the FinnGen database R9(https://r9.risteys.finngen.fi/endpoints/J10_CHRONRHINITIS)
